# Bioactive Dehydrotyrosyl and Dehydrodopyl Compounds of Marine Origin

**DOI:** 10.3390/md8122906

**Published:** 2010-12-06

**Authors:** Manickam Sugumaran, William E. Robinson

**Affiliations:** 1 Department of Biology, University of Massachusetts Boston, Boston, MA 02125, USA; 2 Environmental, Earth and Ocean Sciences Department, University of Massachusetts Boston, Boston, MA 02125, USA; E-Mail: william.robinson@umb.edu

**Keywords:** marine alkaloids, bioactive peptides, dehydrotyrosine, dehydrodopa, dehydrotopa

## Abstract

The amino acid, tyrosine, and its hydroxylated product, 3,4-dihydroxyphenylalanine (dopa), plays an important role in the biogenesis of a number of potentially important bioactive molecules in marine organisms. Interestingly, several of these tyrosyl and dopa-containing compounds possess dehydro groups in their side chains. Examples span the range from simple dehydrotyrosine and dehydrodopamines to complex metabolic products, including peptides and polycyclic alkaloids. Based on structural information, these compounds can be subdivided into five categories: (a) Simple dehydrotyrosine and dehydrotyramine containing molecules; (b) simple dehydrodopa derivatives; (c) peptidyl dehydrotyrosine and dehydrodopa derivatives; (d) multiple dehydrodopa containing compounds; and (e) polycyclic condensed dehydrodopa derivatives. These molecules possess a wide range of biological activities that include (but are not limited to) antitumor activity, antibiotic activity, cytotoxicity, antioxidant activity, multidrug resistance reversal, cell division inhibition, immunomodulatory activity, HIV-integrase inhibition, anti-viral, and anti-feeding (or feeding deterrent) activity. This review summarizes the structure, distribution, possible biosynthetic origin, and biological activity, of the five categories of dehydrotyrosine and dehydrodopa containing compounds.

## 1. Introduction

The origin of almost all antibiotics used today can be traced back to naturally occurring compounds isolated from bacteria and fungi. While the majority of our current antibiotics are considered “semisynthetic” (*i.e.*, modified versions of natural products that are synthetically mass produced), there is only a limited number of totally man-made antibiotic compounds that have proven to be effective in combating bacterial pathogens, such as the broad-spectrum quinolones and sulfonamides, and the Gram-positive targeted oxazolidinones [1]. Because of this, the search for new antibiotics still relies initially on the identification of natural products. Moreover, development of bacterial resistance to a large number of the currently prescribed antibiotics, particularly multi-drug resistance (MDR), has caused serious concern among health care professionals [2,3]. Many experts agree that the lack of development of new antibiotics to replace the ones that are currently ineffective is currently a major threat to human health, and in the years to come. Therefore, there is an urgent need to develop new antibiotics, especially for controlling MDR bacteria that are often found in hospitals and nursing homes located in high population density areas. More broadly, there is also a pressing need for new anti-fungal, anti-viral and anti-tumor compounds.

Marine organisms seem to be a particularly likely source of new antimicrobial and antitumor compounds. Although over 70% of the Earth’s surface is covered by oceans, only about 15% of the approximately 1.8 to 2.0 million species that have currently been named, live in the marine environment [4]. In contrast, biodiversity appears to be far greater in the oceans than on land, since the oceans currently support 32 phyla, whereas only 12 phyla are present on land [4]. Marine waters are therefore a potentially rich source of undiscovered natural products. Marine metabolites and other natural products have to work in saline environments, thus, to some extent, pre-adapting them to human physiological conditions. Nevertheless, their aqueous environment causes rapid dilution, which could greatly reduce their effectiveness. To avoid such dilution problems, many aquatic organisms have evolved efficient and more potent metabolites that exhibit higher toxicity/biological activity even at extremely low concentrations. Hence, marine metabolites could potentially work in physiological saline at concentrations much below the levels needed by compounds of terrestrial origin.

Within the marine environment, marine invertebrates should prove to be a rich source of bioactive compounds. Over 95% of all animals are invertebrates, with over 33 invertebrate phyla [5]. Nevertheless, over 90% of the marine invertebrate species are yet to be described [5]. We would argue that the diversity of invertebrate community structure, organism-to-organism interactions, intricate food webs (predator-prey as well as competition), broad range of reproductive strategies, and host-parasite relationships would make invertebrates a potentially rich source of bioactive compounds. Lacking the complicated immune system of higher animals, marine invertebrates successfully manage to evade and fight infection using a plethora of host defense mechanisms including the use of antibiotic peptides. With such advantages, it is clear that marine invertebrate compounds deserve more attention in the development of newer and more effective antibiotics and other important bioactive compounds.

However, a large-scale, animal collection, tissue homogenization and activity-screening approach to examining possible bioactive compounds in invertebrates is a poor strategy, and is unlikely to be repeatedly successful. René Dubos, credited with the discovery of the first naturally occurring antibiotic gramicidin, was also the first to advocate for a deliberate, systematic approach for searching for antibacterial compounds [6]. The conventional ways to find new antibiotic compounds, although more systematic than the “shotgun” approach, rely on screening techniques employing libraries of both synthetic and naturally occurring compounds. Yet such screening techniques have not generated the much-needed arsenal of chemicals to combat different disease-causing bacteria. We advocate a different systematic strategy—use known structural data on specific groups of bioactive compounds that have been isolated from marine invertebrates to identify likely antibiotics, and then use chemical modification and synthesis to enhance antibiotic potency. Unfortunately, current knowledge of groups of bioactive compounds is patchy and incomplete, thereby limiting the number of systematic studies that can be sustained.

One particularly rich group of bioactive compounds is the dopa/topa-containing compounds in marine invertebrates (and more specifically, dehydrotyrosyl/dopa compounds). Even a cursory glance at the literature of compounds possessing dehydrotyrosyl/dopyl units reveals that these compounds are widely and uniquely distributed in marine invertebrates, possess unusual biological properties, and often exhibit antibiotic activities. Such compounds have been found in a very diverse (and apparently unrelated) number of phyla, including the Porifera (sponges), Cnidaria (coelenterates), Mollusca (bivalves), Arthropoda (insects) and Urochordata (ascidians). This latter group is of particular interest to us. Ascidians (also called tunicates or simply sea squirts; subphylum Tunicata, Class Ascidiacea) are sessile marine filter-feeding invertebrates that exhibit features in their free-swimming larval stage that are characteristic of the vertebrates, including gill slits, notocord and dorsal nerve cord, yet are considered to be a highly divergent offshoot from the other members of the Chordata [7]. As we will discuss later, a number of species of tunicates contain various, low molecular weight, dopa- or topa-containing peptides (or modified peptides) in their blood cells or other body tissues including styelins from *Styela clava* [8], plicatamide from *Styela plicata* [9], halocyamines from *Halocynthia roretzi* [10], lamellarins from *Didemnum chartaceum* [11], ferreascidin from *Pyura stolonifera* [12] and tunichromes from eleven species of tunicates [13–19]. Although there is a wealth of information on invertebrate bioactive compounds, to date these data have been stumbled upon (a stochastic process) rather than systematically investigated (a scientific process). Our aim is to review what is known about this group of compounds, highlight chemical relationships within the group, and suggest promising avenues for future research. This is the first step in the approach we advocate to systematically search for new dehydrotyrosyl/dopyl compounds in marine species to add to the existing list of potentially new antibiotic compounds to tackle the multiple drug-resistance problem. Adding the structures of newly discovered dehydrotyrosyl/dopyl compounds to the list of currently known compounds will better enable researchers to then address the structure-activity relationships.

Dopa- and topa-containing compounds often possess dehydro groups in their side chains. Examples span the range from simple dehydrotyrosine and dehydrodopamines to complex metabolic products, including peptides and polycyclic alkaloids. Structural information allows these compounds to be divided into five categories: (a) simple dehydrotyrosine- and dehydrotyramine-containing compounds; (b) simple dehydrodopa derivatives; (c) simple peptidyl dehydrotyrosine and dehydrodopa derivatives; (d) multiple dehydrodopa-containing compounds; and (e) polycyclic condensed dehydrodopa derivatives. We will address each of these in turn.

## 2. Simple Dehydrotyrosine Derivatives: Tuberine, Erbastatin, p-Hydroxycinnamaldehyde, Lasoidine, Botryllamide, Hamigeramine, Hamigeroxalamic Acid, Tridentatols, Anthosamines, Cyclo-Arg-Dehydrotyrosine and Rigidins

**Figure 1:**
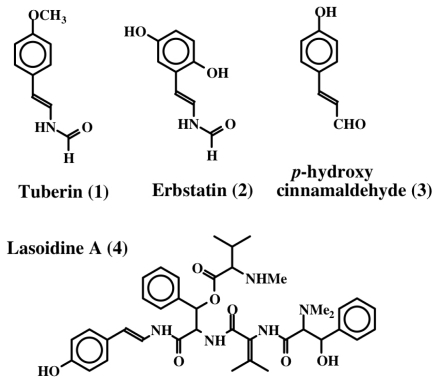
Structures of some simple dehydro compounds.

**Figure 2:**
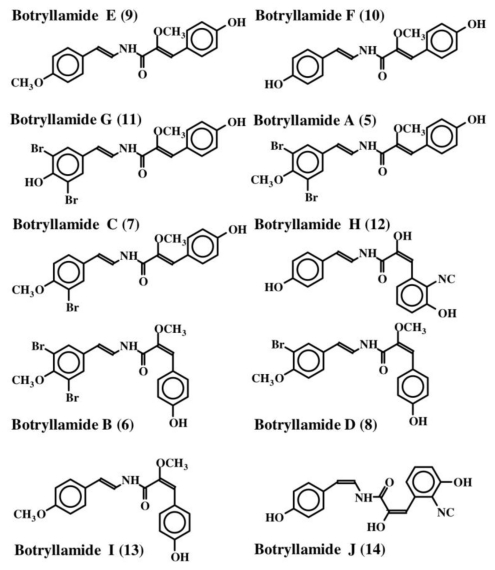
Structures of botryllamides.

The first dehydrotyrosine derivative that was shown to possess biological activity was tuberine [20]. Tuberine (**1**) possessed mild antibiotic properties. The related erbstatin (**2**) was isolated by Umezawa *et al.* [21] (Figure 1) from the culture broth of *Streptomyces amnkusaensis*. These authors demonstrated its potential to inhibit tyrosine-specific protein kinase activity. Leem *et al.* [22] identified *p*-hydroxy cinnamaldehyde (**3**) as an antibacterial substance from the saw fly, *Acantholyda parki*. The tetrapeptide lasoidine A, (**4**) isolated by Marchand *et al.* in 1969 [23] from an African buckthorn plant (family Rhamnaceae), contains a dehydrotyrosine residue. However, the first group of marine compounds possessing dehydrotyrosine unit is botryllamides [24]. They were isolated from two species of ascidians, *Botryllus* sp. from Philippines and *Botryllus schlosseri* from the Great Barrier Reef of Australia. They showed mild cytotoxicity against the human colon cancer cell line HCT-116. Henrich *et al.* [25] used a different species of *Botryllus* (*B. tyreus*) and isolated a number of botryllamides (**5**–**14**) all possessing dehydrotyramine units (Figure 2). These compounds are apparently derived from a tyrosyl tyrosine dipeptide by modification. Botryllamides proved to be effective inhibitors of the ATP binding cassette (ABC) transporter called ABCG2 that is associated with multi drug resistance [25]. They were selective in inhibiting ABCG2 (also known as breast cancer resistant protein, BCRP) and not the two other types of transporters (ABCB1 encoding P-glycoprotein, P-gp; or ABCC1 encoding multidrug associated protein 1, MDR-1) [26].

**Figure 3:**
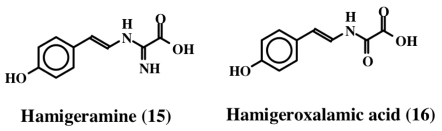
Structures hamigeramine and hemigeroxalamic acid.

From the Mediterranean sponge, *Hamigera hamigera*, Hassan *et al.* [27] isolated two compounds called hamigeramine (**15**) and hamigeroxalamic acid (**16**) whose structures are shown in Figure 3. As is evident, they are simple dehydrotyramine derivates. Due to the low amount of the isolated materials, the authors could not perform quantitative studies, but qualitative studies reveal that these two compounds, along with other unidentified compounds, exhibit fish feeding deterrent activity.

**Figure 4:**
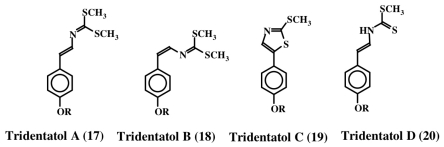
Structures of tridentatols (R = H, **17**–**20**; R = OSO_3_Na are tridentatols E–H, **21**–**24**).

In 1996, Lindquist *et al.* isolated a group of dithiocarbamate related compounds from the marine hydroid, *Tridentata marginata* [28]. They named these compounds tridentatols A, B, and C (**17**, **18** and **19**). In addition, they also isolated a precursor molecule, tridentatol D (**20**), which did not possess any biological activity. The structures of these molecules are shown in Figure 4. Methylation of tridentatol D (**20**) could give rise to tridentatol A (**17**) and cyclization of tridentatol D (**20**) will generate tridentatol C (**19**). Tridentatol B (**18**) is the *cis* isomer of tridentatol A (**17**) that might be formed during the dehydrogenation reaction of a precursor molecule. The *cis* isomer exhibited more potent antioxidant activity against LDL oxidation caused by copper ions than the naturally occurring antioxidant Vitamin E in bioassays [29]. Moreover, these compounds also exhibited deterrent activity as well as protection against solar UV radiation damage [30]. Subsequently, Lindquist [31] isolated five more tridentatol derivates from the same organism and showed that four of them are sulfated derivatives of the tridentatols A–D (**21**–**24**). The sulfated compounds do not possess fish deterrent activity. Upon crushing the marine hydroid, the free forms that possess fish deterrent activity are rapidly released [31].

**Figure 5:**
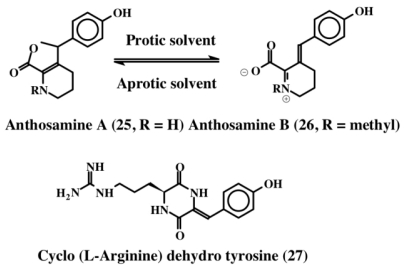
Structures of anthosamines (**25**, **26**) and cyclo arginine dehydrotyrosine (**27**).

During their extended series of studies on the chemical cues that alter larval settlement and metamorphosis, Tsukamoto *et al.* [32] isolated new pipecolate derivatives called anthosamines A (**25**) and B (**26**) and a new diketopiperazine, cyclo-l-arginine-dehydrotyrosine (**27**), from a marine sponge that belong to the Clionidae class, *Anthosigmella* aff. *raromicrosclera* (Figure 5). These compounds possess dehydrotyrosine units and induce larval metamorphosis in ascidian *Halocynthia roretzi.* Anthosamine A (**25**) and B (**26**) exhibit reversible isomerization between amine and iminium ion forms. The amine form predominately occurs in the aprotic solvent while the iminium ion predominates in the protic solvents. The dehydrotyrosine group is conserved in the iminium form and is lost during conversion to the bicyclic amine form (Figure 5).

**Figure 6:**
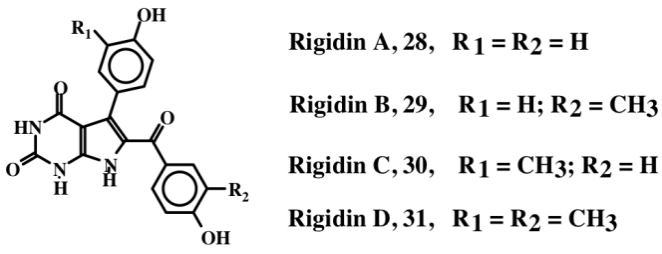
Structures of Rigidins (**28**–**31**).

Rigidin A (**28**, Figure 6), a pyrrolepyrimidine alkaloid containing a dehydrotyramine unit was isolated from the Okinawan marine tunicate, *Eudistoma* cf. *rigida* [33]. This compound exhibited antagonistic activity against calmodulin. The biological activities of the related compounds, rigidin B (**29**), C (**30**) and D (**31**), which were isolated from a different species of tunicate, *Cystodytes* sp., are yet to be determined [34].

## 3. Simple Dehydrodopa Derivatives: 1,2-Dehydro-*N-*acetyldopamine, Narain, Jaspisin, Isojaspisin, Tubastrine, Orthidine and Wondonin A and B, Leucettamines

**Figure 7:**
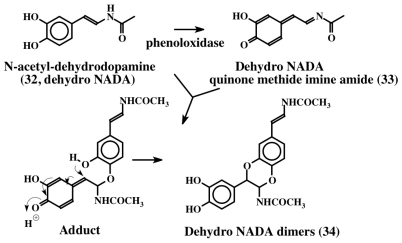
1,2-Dehydro-*N*-acetyldopamine and its dimer formation. Dehydro NADA (**32**) is oxidized by phenoloxidase to quinone methide imine amide (**33**) which adds on to the parent compound forming dimers via a transient intermediate. The reaction yields all four possible stereoisomers (**34**). The reaction of the quinone methide imine amide with *N*-acetyldopamine will produce saturated dimers (**35**, not shown in the figure).

1,2-Dehydro-*N*-acetyldopamine (dehydro NADA, **32**) is one of the most thoroughly studied dehydro compounds. Although this compound is of arthropod origin, given the fact that most of this review is devoted to dehydrodopa derivatives, and that a vast majority of marine metabolites possess the basic structure of this compound, it is important to discuss the biochemistry of this novel compound. Andersen and his associates first isolated dehydro NADA (**32**) from the sclerotized cuticle of locusts and beetles [35,36]. Subsequently, Andersen and Roepstorff isolated this compound as a component of insect cuticle [37]. Since then its chemical synthesis, biogenesis, and potential biological reactions via oxidative transformations have been extensively studied by one of our research laboratories [38–45]. Although this simple compound is very widely used in insects as the major crosslinking agent to make protein-protein adducts, protein-chitin adducts and chitin-chitin adducts, during cuticular hardening process, it is also known to possess antioxidant properties [39]. This compound is very labile and easily undergoes both enzymatic and nonenzymatic oxidation forming dimers. Figure 7 for instance shows the phenoloxidase mediated dimerization of dehydro NADA. Several modifications of this simple structure are widely manifested in marine organisms, as we shall see later.

The cast-off shell (exuviae) of the cicada of *Cryptotympana* sp. (Cicadidae) is used as a crude Chinese traditional medicine called “Zentai”, which is used as an antifebrile, a spasmolytic and as an antiphlogistic. It is also known to possess sedative and sympathetic ganglionic blocking activities. Since insect cuticle is mostly made of polymeric proteins and chitin and to lesser extent catecholamines, the suspected bioactive compound is most likely the catecholamine derivative. Accordingly, several groups of workers have isolated bioactive catecholamine compounds from insect cuticle.

Noda *et al.* [46] first isolated a mixture of saturated dehydro NADA dimers (**35**) as the principle component of the drug. Following this work, Tada *et al.* [47] isolated the same compounds from a different species (*Cryptotympana tuslulata*) and showed that they possessed strong tyrosinase inhibitory activity and could be used as potential cosmetic skin whitening agents. Subsequently, Xu *et al.* [48] isolated both the saturated (**35**) and the unsaturated dimers (**34**) from the exuviae of cicadae available from their region. The unsaturated dimer (**34**) was far more effective in inhibiting reactive oxygen species (ROS) generation, inducible nitric oxide (NO) production and nuclear factor NF-kB activity in biological assays. This compound also induced pro-inflammatory molecules such as inducible nitric oxide synthase (iNOS), interleukin (IL)-6, tumor necrosis factor (TNF)-a and cyclooxygnease (COX)-2 in lipopolysaccharide (LPS) induced cells [48]. Thus both the saturated and unsaturated dimers seem to possess antioxidant and anti-inflammatory properties. However, the “dehydrodopa” derivatives isolated from marine organisms possess even more interesting properties.

**Figure 8:**
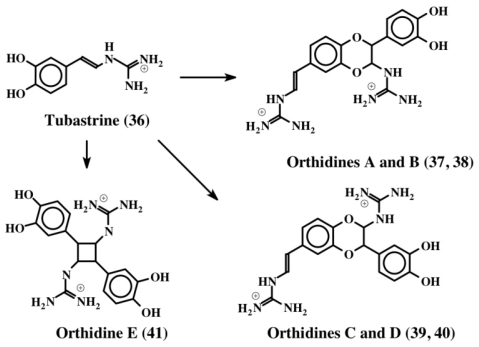
Structures of tubastrine and orthidines. Orthidines (**37**–**41**) are dimers of tubastrine (**36**) formed by a similar mechanism shown in Figure 7 for the dimerization of dehydro NADA (**32**).

The first compound to be identified in this group is tubastrine [49]. It is a guanido dihydroxystyrene compound (**36**) whose structure is shown in Figure 8. It was isolated from the bright orange-red coral, *Tubastrea aurea* in a polar extract, which also possessed antiviral activity against herpes simplex virus and vesicular stomatitis virus. Subsequently, Sperry and Crews [50] isolated this compound along with its saturated analog from the marine sponge, *Petrosia* cf. *contignata.* In a bioassay directed survey, Barenbrock and Kock [51] identified tubastrine (**36**) as the active compound from several ascidians that showed inhibition of epidermal growth factor receptor using a protein tyrosine kinase assay. Tubastrine was isolated as the principle inhibitory compound from the ascidian, *Dendrodoa grossularia* [51]. Recently, Pearce *et al.* [52] identified tubastrine (**36**) and a number of its dimeric products called orthidines (**37**–**41**), from the ascidian, *Aplidium orthium* (Figure 8).

The four benzodioxan dimers (orthidines A–D; **37**–**40**) are the same kind that were characterized for dehydro NADA (**32**). The cyclobutane ring containing dimer (orthidine E, **41**) is unique and has not been identified in any other system so far. Tubastrine (**36**) and its dimers (**37**–**41**) exhibited *in vivo* and *in vitro* anti-inflammatory activities. These compounds drastically reduced the superoxide production by neutrophils *in vivo* in a murine model of gouty inflammation. Accordingly the *in vitro* production of superoxide by PMA-stimulated human neutrophils was inhibited by tubastrine analogs in a dose dependent fashion with an IC_50_ value ranging from 10–36 μM [52].

**Figure 9:**
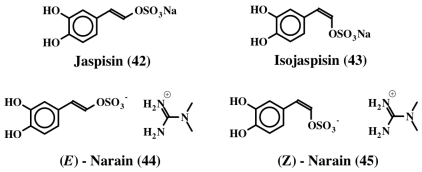
Structures of Jaspisin related compounds.

Jaspisin (**42**) was another dehydrodopa related compound that was isolated from the marine sponge, *Jaspis* sp. [53]. It is the sodium salt of (*E*)-5,6-dihydroxystyryl sulfate (Figure 9) and inhibited the fertilization of sea urchin gametes by blocking the exoplasmic membrane fusion with a minimum inhibition concentration of about 60 μM. The same authors also isolated and reported [54] the structure of the (*Z*) isomer, isojaspisin (**43**, Figure 9). Jaspisin (**42**) inhibited the fusion of acrosome reacted sperm with the plasma membrane of the egg [55]. However, it did not inhibit the acrosome reaction in sperm or the egg cortical reaction. It proved to be a powerful antihatching substance inhibiting the hatching enzyme from sea urchin, a zinc dependent metaloendoprotease [55].

**Figure 10:**
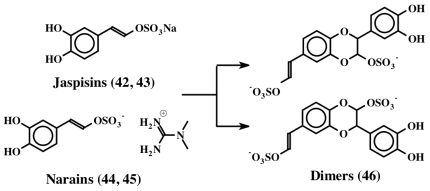
Dimerization of jaspisin.

**Figure 11:**
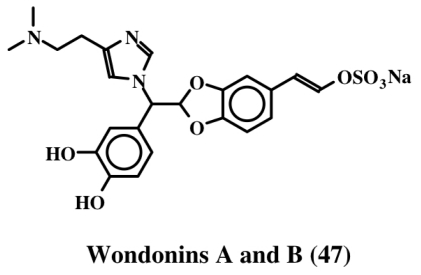
Structure of wondonins.

Narains (*E* and *Z* isomers, **44**, **45**) are *N*,*N*-dimethylguanidium salts of dihydroxystyryl sulfates (Figure 9) isolated from a *Jaspis* sp. which induce larval metamorphosis in the ascidian *Halocynthia roretzi* [56]. Analogous to the dimers of dehydro NADA (**32**) and tubastrine (**36**), narains (and jaspisins) also form dimers (Figure 10). These dimers (**46**) also induce larval metamorphosis in ascidians *Halocynthia roretzi* and *Ciona savignyi* [57]. A slightly different dimer which has lost one sulfate unit was isolated from an association of the sponges, *Poecillastra wondoensis* and *Jaspis* sp., and named wondonins A and B (**47**, Figure 11). These compounds exhibited antiangiogenic activity against human umbilical vein endothelial cells [58].

**Figure 12:**
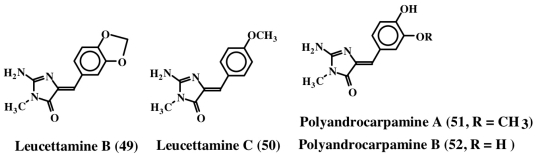
Structures of polyandrocarpamines and leucettamines.

Three new imidazole alkaloids (**48**, **49** and **50**) were isolated from a Palauan sponge, *Leucetta microraphis* [59]. Although leucettamine B (**49**) was inactive, leucettamine A (**48**, structure not shown), a compound possessing extra benzylic group, showed potent inhibition of leukotriene B4 receptor binding activity with a Ki of 1.3 μM. In contrast, leucettamine C (**50**) exhibited only a mild antibiotic activity against *Eschericia coli*, *Staphylococcus aureus*, *Bacillus subtilis* and *Candida albicans* [60]. Recently, several derivatives of leucettamine B (**49**) were synthesized and shown to inhibit protein kinase activity [61]. Polyandrocarpamine A and B (**51**, **52**), are related to leucettamines. They were isolated from a Fijian ascidian [62]. Polyandrocarpamine A (**51**) exhibits selective cytotoxicity against central nervous system cell line (SF 268) with a GI value of about 65 μM [63]. The structures of these molecules are shown in Figure 12.

## 4. Simple Peptidyl Dehydrotyrosine and Dehydrodopa Derivatives: Clionamide, Morulin PM, and Plicatamide

The first peptide possessing a single dehydrodopa unit to be isolated from any organism was that of clionamide (**53**) extracted from the sponge *Cliona celata* [64]. It was shown to have the structure 6-bromotryptophan-dehydrotopamine (**53**). It exhibited only a mild antibiotic activity in comparison with the potency of the crude *Cliona* extracts. Subsequent studies provided a number of additional peptidyl dehydrodopa compounds that may explain this discrepancy (discussed in the next two sections).

Morulin PM (**54**) was isolated as an unusual post translationally modified peptide possessing both topa and 6-bromotryptophan residues from the morula cells of the vanadium accumulating ascidian, *Phallusia mammillata* by Taylor *et al.* [65]. Structural studies revealed that the *C*-terminal residue of the peptide possess a dehydrotopamine residue. Presence of both dehydrotopamine unit and 6-bromotryptophan in the same peptide indicates the likelihood of antibacterial activity for this compound. However, test results have not supported this hypothesis so far, although more systematic studies may resolve this issue. In this regard, it is interesting to note that the octapeptide called plicatamide (**55**), possessing the structure Phe-Phe-His-Leu-His-Phe-His-dehydrodopamine, was isolated from the tunicate, *Styela plicata*, and shown to possess powerful antibiotic activity against *Staphylococcus aureus* [9,66]. Both wild type and methicillin resistant *S. aureus* strains exhibited massive efflux of potassium ions upon exposure to plicatamide. Within seconds bacterial strains exposed to plicatamide ceased to consume molecular oxygen and became nonviable. Plicatamide (**55**) was also shown to be a potent hemolytic agent against human erythrocytes but had no effect on ovine erythrocytes [66]. Its relatively small size, combined with the rapid impact on ion channels [66], make plicatamide a powerful lead compound to develop potential antibiotic compounds in future.

While not a member of the peptidyl dehydrotyrosine and dehydrodopa derivatives category of compounds, styelins are a small group of dopa-containing peptides isolated from ascidians (*Styela* sp.) that exhibit antimicrobial activities against several kinds of bacteria and yeasts, and show cytotoxic activities against eukaryotic cells as well [67]. The hemocytes of *Styela clava* possess a 32 amino acid antimicrobial peptide, called styelin D, having multiple dopa units (but no dehydrodopa units) [8,67]. Styelin D inhibited the growth of both Gram-positive and Gram-negative bacteria and exhibited hemolytic and cytotoxic properties against eukaryotic cells. A much smaller tetra peptide possessing both dehydrotryptophan and dopa units, has been isolated and characterized from the hemocytes of the solitary ascidian, *Halocynthia roretzi* [10,68]. The remaining group of styelins (A, B, C and E), all isolated from *Styela clava*, form pores in bacteria causing leaching of nutrients and eventual cell death [67]. Two other dopa- and topa-containing peptides, halocyamines [10] and ferreascidin [12], have also been isolated from tunicates.

## 5. Peptides with Multiple Dehydrodopa Units: Tunichromes, Polyphenolic Protein and Celenamides

**Figure 13:**
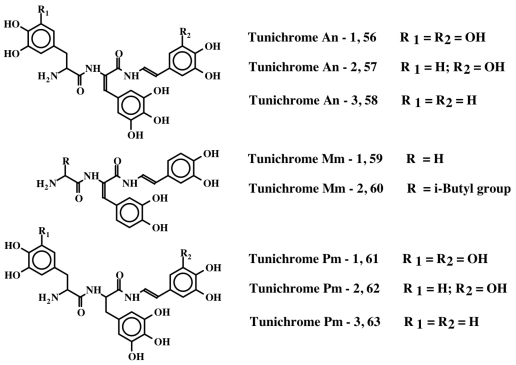
Structures of some tunichromes.

Tunichromes were the first members of this group to be isolated. Tunichromes have been isolated solely from ascidians. They are small, modified peptides that have been extracted and characterized from blood cell lysates and tissues of the phlebobranch *Ascidia nigra* (**An-1**, **An-2**, **An-3; 56**, **57**, **58**) [13–16], the stolidobranch *Molgula manhattensis* (**Mm-1**, **Mm-2; 59**, **60**) [16], and the phlebobranch *Phallusia mammillata* (**Pm-1**, **Pm-2**, **Pm-3; 61**, **62**, **63)** [17]. In addition, they have been identified by thin layer chromatography of hemocyte extracts in seven additional Phlebobranch species [18]. The structures of these peptidyl derivatives are presented in Figure 13 and Table 1. More recently, a modified pentapeptide tunichrome (**Sp-1; 64**) has been isolated from the hemocytes of the stolidobranch ascidian *Styela plicata* [19].

Common to all tunichromes is the presence of dehydrodopamine units. Many other peptides from tunicates, though not part of the tunichrome family of compounds, also possess dehydrodopamine units (for structure, refer to Table 1). In addition, two groups of peptides isolated from the sponge *Cliona celata*, the bromotryptophan containing dipeptide clionamide (**53**, discussed in section 4) [64] and the tri- and tetrapeptide celenamides (**65**–**69**) [69–71], most of which contain dehydrodopamine/topamine units.

Several antibacterial peptides including styelins, clavanins, halocyamines and plicatamide have been isolated from ascidians. Many of them possess dopyl units [8,10,66–68,72–76]. Due to extreme instability and low availability, tunichromes have not been generally tested for antibiotic properties. Preliminary results, using a novel extraction protocol that maintains stability of the native compounds [77], indicate that tunichrome mixtures isolated from *Ascidia nigra*, exhibit antimicrobial activity against Gram-negative bacteria [78]. Future studies will confirm whether this antibiotic activity is widespread among the multiple dehydrodopa-containing peptides.

**Figure 14:**
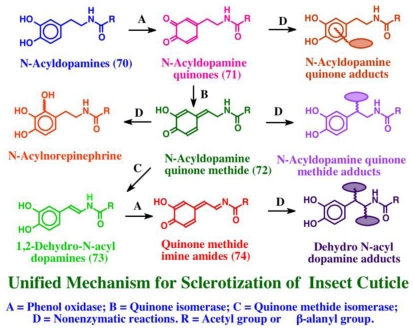
Mechanism associated with the sclerotization of insect cuticle.

Asidians possessing tunichromes accumulate large amounts of trace metals such as vanadium and/or iron [16,79–81]. In *Ascidia ahodori*, vanadium comprised as much as 2% of the dry weight of the hemocytes [82]. It was originally postulated that tunichromes were involved in metal accumulation [17,79–81]. However, this hypothesis is not in harmony with the finding that the metals are located in separate blood cells from the tunichromes [83]. Studies carried out in our laboratory suggest that tunichromes are associated either with cross-linking, wound healing and/or tunic formation [78]. In this regard, it is important to point out that the monomeric unit found in tunichromes, namely dehydrodopamine, is the very same compound that is involved in the sclerotization and hardening of insect cuticle [44,45]. During cuticular hardening in insects and most arthropods, structural proteins and carbohydrate polymer chitin are cross-linked and rendered insoluble by the covalent interaction with reactive intermediates generated from sclerotizing precursors such as *N*-acetyldopamine and *N*-β-alanyldopamine, generally called *N*-acyldopamines (**70**). These two precursors are enzymatically converted to quinones (**71**), quinone methides (**72**) and dehydro *N*-acyldopamines (**73**) prior to cross-linking [44,45]. Upon enzymatic oxidation, dehydro *N*-acyldopamines (**73**) generate quinone methide imine amides (**74**) as initial two-electron oxidation products. These reactive intermediates then react non-enzymatically with chitin and protein to form protein-protein, protein-chitin and chitin-chitin adducts and cross-links that are necessary for hardening and gluing the cuticle. The course of sclerotization reactions employed by insects is depicted in Figure 14.

Marine bivalves use a similar multiple dopyl-containing protein called polyphenolic protein (MW 130 kDa) under oxidative conditions to crosslink byssal threads in order to attach themselves and bind to solid surfaces [84–86]. Polyphenolic protein is composed of a decapeptide sequence containing a tyrosine and a dopa, which is repeated as many as 75 times [84]. With more than one dehydrodopa and/or dehydrodopamine units, tunichromes are likely to exhibit similar reactions, and hence may be involved in tunic formation and wound healing, which are similar to the processes involved in the hardening of insect cuticle [78].

## 6. Polycyclic Condensed Dehydrodopa Derivatives: Halitulin, Storniamides, Purpurones, Ningalins, Baculiferins, Lamellarin, Dictyodendrins, Polycitrins, Polycitones and Lukainols

**Figure 15:**
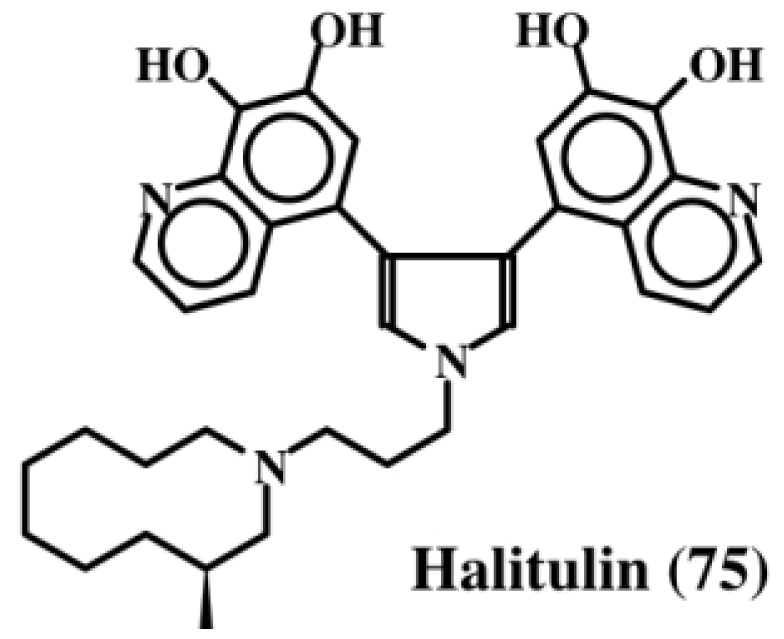
Structure of halitulin.

Kashman *et al.* [87], who have examined the chemistry and bioactivity of a number of marine sponges, found that the extract of the sponge, *Haliclona tulearensis* (family Chalinidae) possessed cytotoxic activity and isolated an active component, which they named halitulin (**75**, Figure 15). This compound exhibited an IC_50_ value of: (a) 25 μg/mL against P-388 murine leukemia cell line; (b) 12 μg/mL against A-549 human lung carcinoma cells; (c) 12 μg/mL against HT-29 human colon carcinoma cells; and (d) 25 μg/mL against MEL-28 human melanoma cell line. In addition to possessing a pyrrole entity, halitulin also, interestingly enough, possess fused dehydrodopamine units [87].

**Figure 16:**
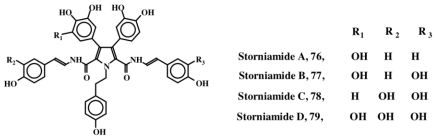
Structures of Storniamides.

Marine sponges of the genus *Cliona* (family Clionidae) have turned out to be rich sources of dehydrodopa containing compounds (see previous discussions in Sections 4 and 5). Clionidae are burrowing organisms that live on calcareous substrates such as coral shells and rocks. They cause serious damage to coral beds and oyster reefs, since they can bore into calcareous structures easily. As mentioned earlier, clionamide (**53**, Section 4) and celenamides (**65**–**69**, Section 5) were two groups of compounds that were initially isolated from *Cliona celata.* Subsequently Palermo *et al.* [88] isolated storniamides A–D (**76**–**79**, Figure 16) through a bioassay guided fractionation procedure, and identified them to be a new group of pyrrole alkaloids exhibiting antibiotic activity against several Gram-positive bacteria. Bogar’s group [89] further studied these compounds and showed that they possess cytotoxicity against leukemia cell lines. In addition, they also identified multi drug resistance (MDR) reversal activity. Furstner *et al.* [90] also reported that storniamide A (**76**) possesses a DNA cleaving property, in addition to MDR reversal activity. Storniamides are probably biosynthesized by free radical coupling of the dipeptide, tyr-tyr followed by oxidative decarboxylation of the *C-*terminal carboxylic group. Dehydrotyrosyl-dehydrotyramine dimeric peptide thus formed, upon condensation with *p*-hydroxyphenylacetaldehyde (produced by monoamine oxidation of tyramine) will produce the basic stroniamide skeleton.

**Figure 17:**
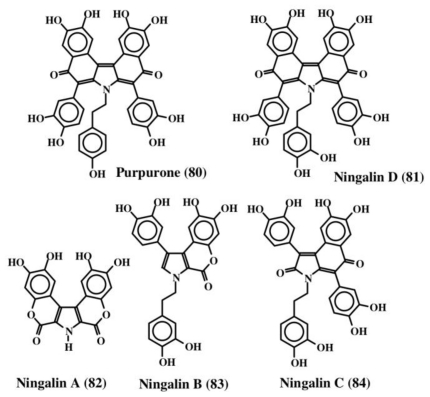
Structures of purpurone and ningalins.

A purple colored pentacylic dehydrodopa derivative called purpurone (**80**, Figure 17), was isolated from the Indopacific sponge of the genus *Iotrochota*, which was shown to inhibit ATP citrate lyase activity with an IC_50_ value of 25 mg/mL [91]. ATP citrate lyase is essential for the synthesis of acetyl CoA and hence reduction in the production of acetyl CoA could drastically affect both lipogenesis and choleterogenesis. Therefore, purpurone (**80**) could be a valuable drug to lower low-density lipoproteins, which are produced from fatty acid and cholesterol. Since purpurone was liberated by acid hydrolysis, it is probably present as a sugar and/or protein conjugate. Purpurone (**80**) has also been isolated from a south sea marine sponge of the same genus, *Iotrochota*, by a bioactivity-guided protocol aimed at identifying antioxidant compounds by Liu *et al.* [92].

A hydroxylated derivative of purpurone was subsequently isolated from a western Australian unidentified ascidian belonging to the genus *Didemnum* and named ningalin D (**81**) [93]. These authors also isolated a number of biogenetically related compounds that were like purpurone from the same organism (ningalins A, B, and C; **82**–**84**, Figure 17). Both ningalins, as well as their derivatives, exhibited marked cytotoxicity against several cancer cell lines. In addition, they also exhibited significant MDR reversal activity at non-cytotoxic concentrations [94,95]. Recently a group of about five different compounds that are derivatives of purpurone and ningalins were isolated from the Chinese marine sponge, *Iotrochota baculifera* and shown to possess anti HIV-1 activity [96]. Since ningalins resemble a number of lamellarins, it was proposed that ningalins might serve as the biosynthetic precursor for a number of lamellarins [93].

**Figure 18:**
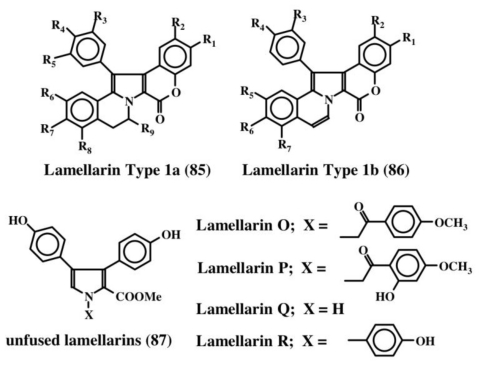
Structures of lamellarins.

Lamellarins are a group of over seventy different polycyclic condensed aromatic compounds [11]. Faulker and his group first reported the isolation and characterization of four lamellarins from the prosobranch mollusc, *Lamellaria* sp. in 1985 and named them lamellarin A–D [97]. Subsequently, four additional members of this group (E–H) were isolated form the didemnid ascidian *Didemnum chartaceum* [98]. Since then, as many as seventy different, yet structurally closely related, polycyclic aromatic condensed compounds have been isolated from a variety of marine organism possessing a wide range of biological activities that include (yet not limited to) cytotoxicity, antibiotic activity, antitumor activity, antioxidant activity, MDR reversal activity, HIV integrase inhibition, human aldose reductase inhibition, cell division inhibition, immunomodulatory activity, and feeding deterrent activity [11]. Since an excellent review article is available solely on all lamellarins [11], it is unnecessary to review the structure function aspect of lamellarins again here. Lamellarins fall broadly under three structural categories shown in Figure 18. The majority of the lamellarins possess either Type 1a or 1b structures (**85**, **86**). These compounds contain the dehydrodopamine unit. The last structure (*i.e.*, unfused lamellarins, **87**) lacks the dehydrodopamine unit but possesses instead dehydrotyramine unit. Only a few compounds (about four) belong to this category. Interestingly, a number of related compounds possessing only dehydrotyramine units are also identified from marine organisms and the rest of this section deals with such compounds. Even though they are not dehydrodopamine containing molecules, since they are all polycyclic condensed molecules similar to lamellarins, they are listed here for simplicity’s sake.

**Figure 19:**
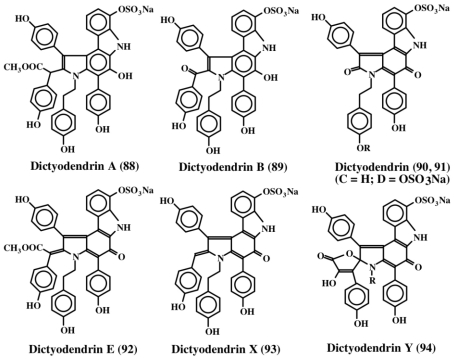
Structures of dictyodendrins.

Warabi *et al.* [99] isolated five compounds, which they named dictyodendrins (A–E; **88**–**92**), from the sponge *Dictyodendrilla verongiformis*, as potent inhibitors of telomerase. Structures of some of these compounds along with two related compounds (**93**, **94**) [100] are shown in Figure 19. Telomerase, which adds repeats of TTAGGG units called telomeres, to the 3′-end of chromosomes, is inhibited by these compounds at 50 μg/mL level. Since telomerase is found nearly in 90% of cancer cells but not in any significant amount in normal cells, this finding could pave the way to the development of new kinds of anticancer drugs.

**Figure 20:**
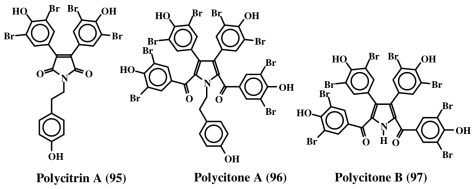
Structure of polycitrin A (**95**) and polycitones (**96**, **97**).

Rudi *et al.* [101] isolated polycitrin A (**95**) and polycitone A (**96**) (Figure 20) from the ascidian, *Polycitor* sp. in 1994 and showed that the penta-*O*-methyl derivative of polycitone A (**96**) could inhibit SV40 transformed fibroblast cells at concentrations as low as 10 μg/mL. Subsequently, this group was able to demonstrate the inhibition of retroviral reverse transcriptases (isolated from human immunodeficiency virus, murine leukemia virus, and mouse mammary tumor virus) as well as cellular DNA polymerase (DNA polymerase B and *E. coli* DNA polymerase I) activities by polycitone A (**96**) [102]. Furthermore, they also isolated polycitone B (**97**) and prepolycitrin A from the ascidian, *Polycitor africanus* [103].

**Figure 21:**
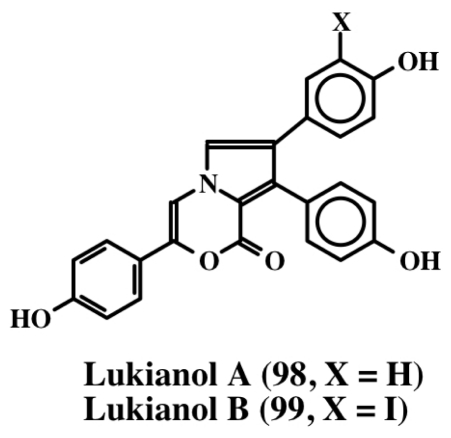
Structure of lukianols.

From the extracts of a Pacific tunicate, Scheuer *et al.* [104] isolated lukianol A and B (**98**, **99**; Figure 21). Both compounds exhibited toxicity against a human epidermatoid carcinoma cell line. Lukianol A (**98**) possessed a minimum inhibitory concentration of 1 μg/mL and lukianol B (**99**) possessed a minimum inhibitory concentration of 100 μg/mL. Subsequently, Boger *et al.* [89] synthesized and tested lukianol A (**98**) and other related synthetic compounds on a group of cancer cell lines. The results indicated that these compounds possessed IC_50_ values ranging from 1–20 μM. Although lukianol A did not possess any MDR reversal activity on the cellular P-glycoprotein efflux pump, one of its synthetic precursors showed a reasonable MDR reversal activity at non-cytotoxic concentrations. In this regard, it is interesting to note that the pyrroles possessing phenolic substituents seem to be associated with cytotoxicity, whereas phenolic ethers tend to exhibit MDR reversal activity. Gupton’s group [105,106] synthesized one of the key intermediate of lukianol A (**98**) and demonstrated its potent cytotoxicity against a number of cancer cell lines with ED_50_ values ranging from of 3–20 μM. In the lymphocytic leukemia cell lines, this compound also inhibited both DNA synthesis and protein synthesis by inhibiting a number of key enzymes such as DNA polymerase a, some RNA polymerases, ribonucleotide reductase, dihydrofolate reductase, phosphoribosyl pyrophosphate transferase and IMP dehydrogenase [105,106]. Even compounds with only one aryl group showed cytotoxicity at the same level as the diaryl pyrrole [105,106].

## 7. Biogenesis of Dehydro Compounds

**Figure 22:**
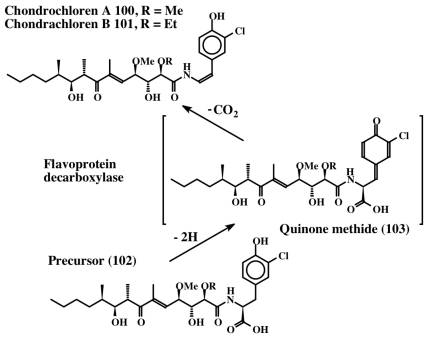
Formation of dehydrotyramine.

From the foregoing discussion it is evident that unusually large numbers of dehydrotyrosine and dehydrodopa compounds are biosynthesized in marine organisms. In spite of accumulation of such a vast knowledge on the isolation and characterization of a plethora of these molecules, the information on their biosynthetic pathways is pretty scarce. Therefore, one can only hypothesize on their biosynthetic mechanism(s), based on what little is known about the biosynthesis of dehydrotyrosine and dehydrodopa compounds. From the structures, it is evident that these compounds arise by the introduction of the double bond in the side chain of the saturated derivatives of the parent compounds *viz*., tyrosine and dopa. Examination of the biosynthetic literature on the fate of tyrosine and dopa reveals that two different routes make dehydrotyrosine and dehydrodopa derivatives. The introduction of the double bond in peptidyl tyrosine units can be achieved by the action of the recently identified enzyme, tyrosine side chain oxidase [107]. This enzyme, first identified in the biosynthesis of the antibiotic compound, chondrochloren (**100**, **101**) produced by *Chondromyces crocatus*, causes oxidative decarboxylation of an acyltyrosine peptide (**102**) (synthesized by nonribosomal peptide synthesis) to a dehydrotyramine containing compound (Figure 22).

**Figure 23:**
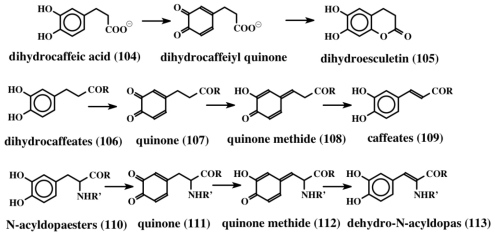
Oxidative transformation of protected dopa compounds. Deaminated dopa (dihydrocaffeic acid), upon oxidation to quinone, undergoes intramolecular cyclization, producing a dihydroesculetin type structure that is prevalent in lamellarins and other compounds (top line). If the carboxyl group is protected, the resultant quinone undergoes isomerization to quinone methide and then produces dehydro compounds (middle line). The same reaction is also exhibited by *N*-acyldopa esters, as shown on the last line.

Isolation and expression of the gene in *E. coli* and examining the gene product revealed that it is a FAD-dependent oxidative decarboxylase [107]. It seems to oxidize tyrosine precursor (**102**) to a transient quinone methide (**103**) by extracting two hydrogen atoms from the 1,6 position (from the phenolic group and the methylene group, as shown in Figure 22). The resultant quinone methide (**103**) spontaneously loses the carboxyl group and undergoes isomerization to a dehydrotyramine-containing compound (**100**, **101**). If the carboxyl group is blocked by acylation, such as peptide bond formation, the carboxyl group will remain intact. Loss of a proton from the neighboring methylene group in such molecules and simultaneous aromatization of the quinone methide nucleus, results in the generation of dehydrotyrosine units. On the other hand, if the carboxyl group is unprotected, it will suffer a rapid and spontaneous nonenzymatic decarboxylation reaction accompanied by the aromatization of the quinone methide nucleus that generates dehydrotyramines. A direct hydroxylation of dehydrotyrosines and dehydrotyramines by a tyrosinase type enzyme can lead to the dehydro derivatives of dopa and dopamine. Alternatively, dehydrodopa and dehydrodopamines can also be made from their saturated analogs by a different route. The amino acid tyrosine is converted to dopa in a number of biological systems by the action of a specific tyrosine hydroxylase as well as by tyrosinase. While tyrosine hydroxylase reaction stops at the production of dopa, the tyrosinase acts further on the resultant dopa generating dopaquinone as the transient two-electron oxidant product. Dopaquinone is highly unstable and undergoes rapid intramolecular cyclization producing leucochrome initially, which gets further oxidized nonenzymatically to produce a series of compounds that finally result in the polymerization and production of melanin pigments [44,45,108]. This reaction, which is useful for the biosynthesis of melanin polymer, is not very helpful for producing dehydro derivatives of dopa and dopamine. Protecting the amino group by acylation deactivates the amino group reactivity and prevents the intramolecular cyclization of protected dopaquinones. Such compounds, upon oxidation by tyrosinase, can generate dehydrodopa derivatives. For instance, the deaminated dopa, dihydrocaffeic acid (**104**), upon oxidation by tyrosinase produces both dihydroesculetin (**105**, a cyclized product) and traces of caffeic acid (desaturated product) as shown in Figure 23 [109]. If the carboxyl group is also prevented from exhibiting intramolecular cyclization reaction—say by amide formation or esterification—the resultant dihydrocaffeate derivates (**106**) solely exhibit side chain desaturation reaction when oxidized by tyrosinase [109,110]. The reaction was shown to occur by the spontaneous isomerization of tyrosinase produced quinone (**107**) to quinone methide (**108**) and subsequent aromatization of the quinone methide with the introduction of the double bond in the side chain leading to the caffeic acid derivatives (**109**) (Figure 23).

Dopa derivatives also exhibit this reaction when both the carboxyl and amino groups are protected by acylation and amide formation. Accordingly, the tyrosinase generated quinones from both *N*-acetyldopa methyl ester and ethyl esters (**110)** exhibit spontaneous isomerization reaction to produce dehydrodopa derivatives (**113**) via quinone (**111**) and quinone methide intermediates (**112**) [111,112]. Thus peptidyl dopa derivatives can be converted to dehydro dopa units by simple tyrosinases that are present ubiquitously in biological systems without the need for any other enzymes. On the other hand, if the carboxyl (or carbonyl) group is absent to assist the quinone-to-quinone methide conversion, as in the case of 1,2-dehydro-*N*-acyldopamine production, there is a need for additional enzymatic catalysts [113–115]. If the carboxyl group in dopa is decarboxylated to dopamine, direct introduction of the double bond into its side chain is rather difficult. However, this reaction is achieved by the use of tyrosinase and quinone isomerases [44,45]. Unprotected simple dopamine will generate only melanin type molecules as mentioned earlier. But if the amino group is protected by acylation, *N*-acyldopamines thus formed, upon oxidation by tyrosine will produce *N*-acyldopamine quinones that are quite stable. We have identified two separate isomerases—one that acts on *N-*acyldopamine quinone producing quinone methide [113,114] and the other, which acts on the resultant quinone methide producing the side chain desaturated product—dehydrodopamine [115]. Thus if the carboxyl group is removed from dopa, one has to have two additional enzymes to cause the isomerization of the quinone and the quinone methide (shown in Figure 14). Finally, dehydrodopamine can also arise from the decarboxylation of amino protected dopaquinone derivatives after quinone methide formation, much like the tyrosyl peptide to dehydrotyramine peptide route discussed above.

Examination of the structures of a number of condensed compounds indicates that coumarin type molecules are also formed routinely in ningalins and lamellarins. This condensation can occur by intramolecular cyclization of *N-*protected dopyl compounds to form the six-membered ring [109]. We have shown such a facile cyclization as early as 1989 in the case of dihydrocaffeic acid (Figure 23 top line). Finally, enzymology of the biosynthetic process of all these naturally occurring compounds is practically untouched and might yield rich details for future investigators. All our attempts to identify the isomerases in ascidians have so far fallen short of confirming their presence in tunicates. More biochemical studies are necessary to throw light on this process.

## 8. Conclusions

Dehydrotyrosyl and dehydrodopyl compounds have been isolated from a broad range of invertebrate phyla (Table 2). These compounds are generally either peptides or small proteins. All have been isolated from organisms that are sedentary as adults. Furthermore, each of these compounds is bioactive to some degree, most likely due to the highly reactive catechol group(s). However, different types of bioactivity have been reported for different compounds, including antibiotic and other defense activities, enzyme inhibition, free radical mediation, wound repair, and structural crosslinking of components of exoskeletons. All of these varied processes can be, in theory, attributed to reactive catechols. Surprisingly, these diverse compounds appear quite common in two widely separated Phyla—the simple metazoan sponges (Phylum Porifera) and, at the opposite extreme, the bilateral eumetazoan deuterostome ascidians (Phylum Chordata, subphylum Urochordata). Examples have also been isolated from intermediate phyla, including the radially symmetrical Phylum Cnidaria (hydroids), and the bilateral protostome Phylum Mollusca (bivalves). Even more surprising, several of the same dopa-containing compounds have been isolated from members of different phyla (Table 2). The simple dehydrodopa compound tubastrine has been found in a sponge, a coelenterate and an ascidian. In the ascidian, not only has the monomeric tubastrine (**36**) been identified, but a dimeric form (named orthidine, **37**–**41**) has also been isolated. In addition, the tri-peptide tunichromes, which are widespread in the ascidians, are structurally similar to the bromotryptophan-containing dipeptide clionamide (**53**) and to the tri- and tetra-peptide celenamides (**65**–**69**), both of which are found in sponges. Finally, the more complex, polycyclic condensed dehydrodopa compound purpurone (**80**) was originally isolated from a sponge, but has also turned up in an ascidian as a hydroxylated derivative, ningalin D (**81**). A second polycyclic dehydrodopa family, the lamellarins, was isolated from both a snail and an ascidian.

Given this widespread distribution of dehydrotyrosyl and dehydrodopyl compounds among the invertebrate phyla, and the discovery of the same compounds in widely separate phyla, it appears that the ability to synthesize this group of compounds arose very early in evolutionary history, and has been retained by a variety of animals because of its selective advantage. It is also possible that bacterial symbionts could form a potential source of these compounds. It is likely that examples of these compounds have been found in sponges, hydroids, bivalves and ascidians simply because scientists happened to have looked for them in these groups. If this is the case, then it is very likely that similar compounds are present in other phyla that have yet to be examined. Since all of the organisms that have been successfully assayed to date are sedentary organism, the search for additional compounds should probably begin with members of the fouling communities, including additional hydroids, sedentary polychaete worms, barnacles, bryozoans, and ectoprocts. In addition, one major phylum that has yet to be tested is the ecdhinoderms (seastars, brittle stars, sea urchins, sea cucumbers, *etc.*).

A careful “deconstruction” of all the compounds mentioned in this review indicates that they all start the same way, perhaps from peptidyltyrosine and/or peptidyldopa units. They are most likely synthesized primarily by two different routes. The first involves post-translational modification of simple peptides obtained by traditional ribosomal synthesis. The second employs nonribosomal peptidyl assembly followed by post-translational modification of the resultant small peptide. Both routes are likely to function. Additional post-translational modifications are necessary to produce the final compounds in many cases. Neither the mechanisms nor the sites of synthesis of these compounds have yet been explored.

As a final note, compiling the list of compounds we have reported on in this review (e.g., Table 2) was not a trivial task, given the morass of common names published in the literature. Nomenclature was not based on structural attributes (e.g., tyrosyl or dopyl based), making literature searches difficult. We could very easily have missed additional related compounds in our searches. We highly recommend that authors carefully consider structurally based nomenclature and use structurally based key words (e.g., dehydrotyrosine, dehydrodopa, *etc.*) when publishing newly discovered natural products.

We have reviewed the widespread occurrence of dehydrotyrosyl and dehydrodopyl containing compounds in marine invertebrate phyla, and have highlighted the structural similarities among them. Furthermore, we have speculated that all of these compounds originate via common pathways. Now that we better understand the interrelationships among these compounds, more in-depth structural comparisons relative to a specific type of bioactivity is now possible. For example, all of these structures should be compared in the context of antimicrobial activity. This will allow us to suggest structural modifications (reactive and nonreactive side groups, hydroxylations, methylations, *etc.*) that could be used to optimize antimicrobial activity. These suggested changes can be tested following synthesis of model compounds in the laboratory. At the same time, we should not avoid a further search for new lead dehydrotyrosyl and dehydrodopyl compounds in additional species of sponges, coelenterates, molluscs and ascidians, as well as in other marine phyla that have so far been overlooked. This will provide us with additional structures to include in our scheme of possible structural similarities.

## Figures and Tables

**Table 1:** Structure of some tunichromes and related compounds.

No.	Compound	Structure	References
1	Tunichrome An-1, **56**	Topa-DeTopa-DeTopamine	[14,15]
2	Tunichrome An-2, **57**	Dopa-DeTopa-DeTopamine	[14,15]
3	Tunichrome An-3, **58**	Dopa-DeTopa-DeDopamine	[14,15]
4	Tunichrome Mm-1, **59**	Gly-DeDopa-DeDopamine	[16]
5	Tunichrome Mm-2, **60**	Leu-DeDopa-DeDopamine	[16]
6	Tunichrome Pm-1, **61**	Topa-Topa-DeTopamine	[17]
7	Tunichrome Pm-2, **62**	Dopa-Topa-DeTopamine	[17]
8	Tunichrome Pm-3, **63**	Dopa-Topa-DeDopamine	[17]
9	Tunichrome Sp-1, **64**	Dopa-Dopa-Gly-Pro-DeDopamine	[19]
10	Plicatamide, **55**	Phe-Phe-His-Leu-His-Phe-His-DeDopamine	[9,66]
11	Morulin Pm, **54**	Polypeptide with 6-BrTrp & DeDopamine	[65]
12	Clionamide, **53**	6-BrTrp-DeTopamine	[64]
13	Celenamide A, **65**	Leu-DeTopa-6-BrTrp-DeDopamine	[69–71]
14	Celenamide B, **66**	Val-DeTopa-6-BrTrp-DeDopamine	[69–71]
15	Celenamide C, **67**	Leu-DeTopa-6-BrTrp-DeTyramine	[69–71]
16	Celenamide D, **68**	Leu-DeTopa-DeTopa-DeDopamine	[69–71]
17	Celenamide E, **69**	DeTopa-6-BrTrp-DeDopamine	[69–71]

**Table 2:** Dehydrotyrosyl and dehydrodopyl compounds found in various groups of marine invertebrates. The five categories are explained in the text. The same compound isolated from different groups of animals is highlighted in red.

Category	Sponges	Coelenterates	Molluscs	Ascidians
1	Hamigeramine (**15**); Hamigeroxalamic acid (**16**);	Tridentatols A–D (**17**–**20**)		Botryllamide (**5**–**14**); Rigidin A–D (**28**–**31**)
	Anthosamine A & B (**25**, **26**);			
	Cyclo-l-arginine-dehydrotyrosine (**27**)			
2	***Tubastrine*** (**36**);	***Tubastrine*** (**36**)		***Tubastrine*** (**36**);
	Jaspisin (**42**);			***Orthidines*** (**37**–**41**);
	Isojaspisin (**43**);			Polyandrocarpamine A & B
	Narains (**44**, **45**);			(**51**, **52**)
	Wondonins (**47**);			
	Leucettamine A–C (**48**–**50**)			
3	Clionamide (**53**)			Morulin PM (**54**);
				Plicatamide (**55**);
				Styelin A–D
4	Celenamides (**65**–**69**)		Polyphenolic protein	Tunichromes (**56**–**64**)
5	***Purpurone*** (**80**);		**Lamellarin**	***Ningalin D*** (**81**);
	Halitulin (**75**);		**A–D**	Ningalin A–C **(82**–**84)**;
	Storniamide A–D (**76**–**79**);			**Lamellarin E**–**H**;
	Dictyodendrin A–E (**88**–**92**)			Polycitrin A (**95**);
				Polycitone A & B (**96**, **97**);
				Lukianol A & B (**98**, **99**)
